# Symptom networks in breast cancer patients during chemotherapy and their impact on daily living status

**DOI:** 10.3389/fonc.2026.1709837

**Published:** 2026-02-27

**Authors:** Yuxuan Zhang, Huoling Pan, Jinyu Zhang, Fengjing Wan, Minqi Ma, Wei Zheng

**Affiliations:** 1 School of Nursing, Shanghai University of Traditional Chinese Medicine, Shanghai, China; 2 School of Nursing, Hainan Medical University, Haikou, China; 3 Department of Breast Diseases, Longhua Hospital Shanghai University of Traditional Chinese Medicine, Shanghai, China

**Keywords:** breast cancer, chemotherapy, daily living status, subgroup network analysis, symptom networks

## Abstract

**Background:**

Few current studies explore the relationships among chemotherapy - related symptoms in breast cancer patients from different perspectives, and the link between symptoms and daily life is unclear.

**Objective:**

To construct a symptom network for breast cancer patients during chemotherapy and explore symptom - symptom relationships from multiple perspectives.

**Methods:**

The Anderson Symptom Inventory was used to collect symptom data and daily - life interference of 480 patients. R software built the symptom network, and edge - weight and centrality difference tests identified core symptom clusters.

**Results:**

The 480 female patients had a mean age of 52.46 years. Symptoms were common during chemotherapy, like fatigue and restless sleep. Fatigue was the core symptom in the overall network, but it varied among groups with different chemotherapy cycles and BMI. Distress had the greatest impact on daily life.

**Conclusion:**

Attention should be paid to psychological and emotional symptoms, and priority symptoms for intervention should be selected based on the symptom network. Future research should develop dynamic symptom networks and centrality index trajectories using longitudinal data.

**Implications for practice:**

Nurses can implement precise symptom management based on the symptom network. Meanwhile, comprehensive assessments of patients’ psychological and emotional problems should be initiated from the early stage of chemotherapy, and symptom management methods should be selected according to the severity of patients’ conditions to improve their psychological and emotional well - being.

## Introduction

Breast cancer is one of the most common cancers globally, with approximately 2.3 million new cases reported annually (1). According to a survey by the World Health Organization (WHO), if current trends remain unchanged, the burden of breast cancer will increase to over 3 million new cases and 1 million deaths annually by 2040, driven solely by population growth and aging (2). In 2024, the China Cancer Center released data on the cancer burden in China for 2022, revealing 357,200 new breast cancer cases, accounting for 7.4% of all cancer cases and 15.6% of all female cancer cases (3).

In the field of breast cancer treatment, chemotherapy is widely recognized as a critical component of multimodal therapy for the majority of patients, supported by robust clinical evidence and guideline recommendations (4). It effectively inhibits the proliferation of tumor cells, reduces the risk of recurrence, prolongs survival, and exhibits significant efficacy in controlling distant metastases (5, 6). Despite these benefits, chemotherapy is accompanied by a range of adverse effects, including nausea, vomiting, pain, fatigue, anxiety, depression, and sleep disturbances (7, 8). Throughout the course of treatment, patients may continue to experience persistent or recurring symptoms, with some even demonstrating cumulative effects as therapeutic interventions progress (9). The symptoms related chemotherapy can have long-term impacts on patients’ physical and psychological well-being, leading to changes in body image and function, as well as impairing social relationships. This significantly disrupts patients’ daily lives, reduces health-related quality of life (10), and may result in treatment delays, interruptions, or increased medical costs (11). For breast cancer patients, effectively managing symptoms during chemotherapy is crucial to alleviate their impact on daily life and improve quality of life.

Qi et al. (12) showed that patients with breast cancer receiving chemotherapy usually experience more than 10 types of symptoms. Fatigue is the most common symptom among breast cancer patients during chemotherapy, with a prevalence ranging from 60% to 90% (13). Considerable efforts have been made to study the universal symptoms (14–16) and co-occurring symptoms (17, 18) during chemotherapy in breast cancer patients in the past 20 years. Understanding how symptoms interact could help prevent their occurrence and progression. However, these studies have individually discussed symptoms (19, 20), overlooking the complex relationships between them. With the advancement of research, scholars have begun to recognize that these symptoms do not exist in isolation but rather cluster together. Based on this, researchers have started to explore symptom clusters (21, 22), with the most commonly reported clusters in breast cancer patients during chemotherapy being the gastrointestinal cluster (nausea-anorexia), the pain-fatigue-sleep disturbance cluster, and the psychological cluster (anxiety-depression-concern-sadness-tension-irritability) (22). This represents a significant step forward from studying symptoms in isolation, but symptom clusters are merely a way to group symptoms and can only provide a broad view of which cancer-related symptoms share similar coexistence mechanisms (23). The interactions between symptoms remain unclear, and due to variations in symptom assessment scales, analytical methods, and the treatment stages of patients, the composition of symptom clusters is not consistent. Targeted intervention based on symptom clusters remains a major challenge (24). Moreover, the status of symptoms within a cluster is not equal. Some symptoms play a central role and have a critical impact on the emergence and maintenance of other symptoms. These symptoms are referred to as “core symptoms,” which are the most strongly correlated with other symptoms and may play a key role in activating them (25). This finding has prompted researchers to further propose the concept of “symptom networks” to describe the complex interactions between symptoms (24). Visualizing and exploring the internal network structure of symptoms within specific populations can help researchers identify core symptoms and the associations between them (26). The identification of core symptoms can provide a better understanding of the symptom burden on cancer patients.

Currently, there is a paucity of research specifically constructing symptom networks for breast cancer patients during chemotherapy. The study by Kim et al. (7) established a symptom network for 250 breast cancer patients undergoing radiotherapy and investigated the relationship between symptoms and quality of life, identifying fatigue as the most central symptom within the network. Zhu et al. (27) explored the multidimensional symptom experiences of cancer patients after initial treatment, including those with lung cancer, colorectal cancer, breast cancer, and thyroid cancer, and found that while fatigue is the most severe symptom among cancer survivors, it is not the most central symptom in the symptom network. Although Liang et al. (28) constructed a symptom network for 468 breast cancer patients during chemotherapy, the focus of their research was on identifying symptom clusters and sentinel symptoms, with no in-depth exploration of the relationships between symptoms within the network. Identifying the complex relationships between symptoms is essential for determining the targets of symptom intervention and developing personalized and precise symptom management strategies. It is necessary to construct a symptom network for breast cancer patients during chemotherapy to aid in better symptom management.

In clinical practice, symptom management is typically carried out based on the incidence and severity of these symptoms. However, in fact, the impact of symptoms on daily life may not be consistent with their incidence and severity (29). Kim et al. (30) conducted a survey on the symptom experiences of cancer patients during chemotherapy and found that the prevalence of symptoms interfering with daily life could be as high as 95.8%. Participating in daily life can be regarded as one of the ultimate goals of rehabilitation (31). Lack of exercise, anxiety, depression, reduced social interaction, and lack of life enjoyment can all reduce patients’ quality of life and affect their health status (32, 33). Compared with breast cancer survivors who have completed initial treatment (27), patients undergoing chemotherapy have a higher incidence of symptoms disturbing their daily life, and the impact of symptoms on patients’ daily life is also greater. Research on psychosocial distress in cancer patients and survivors has demonstrated that psychological issues, such as anxiety and depression, act as core mediating factors linking symptoms to daily functioning. Failure to promptly address these psychosocial concerns not only amplifies patients’ pain perception and exacerbates chemotherapy-related side effects but may also diminish survival rates (34). Additionally, it significantly impairs the daily lives of both patients and their families. Evaluating the interference of symptoms on daily life can provide a more comprehensive understanding of the patients’ overall situation. Few studies have incorporated the interference of symptoms on daily life into network analysis. From the perspective of assessing the relationship between symptoms and patients’ daily - life status, it remains unclear which symptoms are the core symptoms affecting patients’ daily life.

Therefore, the objectives of this study are:

1. To construct symptom networks among breast cancer patients during chemotherapy, identify core symptoms and explore the interrelationships among symptoms.
2. To explore the relationship between symptoms and daily living status from the perspective of symptom networks.

## Methods

### Study design and subjects

This study involves a secondary analysis of data collected from a study on symptom profiles and quality of life in breast cancer patients undergoing chemotherapy. Patients were recruited from three tertiary hospitals in Shanghai, China, between April 2021 and December 2021. Data was collected during the patient’s chemotherapy treatment, with specific chemotherapy cycles documented. Participants had to meet the following inclusion criteria: (1)A confirmed diagnosis of breast cancer and currently undergoing chemotherapy. (2) Age ≥ 18 years. (3) Willingness to participate in the study and the ability to complete questionnaires independently.

Exclusion criteria (1) Patients undergoing their first chemotherapy, as chemotherapy-related symptoms have not yet developed at this stage and their inclusion would yield non-informative zero-value measurements. (2) Patients with recurrent or metastatic disease or severe complications. (3) Patients with communication barriers or clinically diagnosed with other psychiatric disorders. Two researchers jointly reviewed the quality of the questionnaires. A total of 495 questionnaires were distributed, of which 480 were valid, resulting in an effective response rate of 96.97%. This dataset includes 480 breast cancer patients, meeting the symptom network analysis requirement of a sample size 20 to 30 times the number of 19 symptom items (35). Chemotherapy cycles and symptom data were recorded synchronously, capturing multiple symptoms throughout treatment. Symptom variability was substantial across cycles, aligning with the core goal of network analysis: constructing symptom networks that reflect complex intersymptom relationships. All symptom data were continuous and compatible with the EBICglasso model, and the dataset contained rich covariates to support subgroup analyses, with all characteristics fulfilling symptom network analysis requirements.

### Measures

Sociodemographic variables included age, education level, employment status, body mass index (BMI), and specific chemotherapy cycle. These data were used to investigate how sociodemographic factors and chemotherapy cycles influence changes in symptom networks. The severity of cancer-related symptoms was assessed using the symptom severity subscale of the Chinese version of the MD Anderson Symptom Inventory (MDASI-C). This subscale consists of 13 items measuring the severity of symptoms such as fatigue, sleep disturbance, pain, drowsiness, lack of appetite, nausea, vomiting, shortness of breath, numbness, memory problems, dry mouth, distress, and sadness. Each symptom item is rated on a scale from 0 to 10, with higher scores indicating greater symptom severity. A validation study involving 342 Chinese breast cancer patients confirmed the applicability of the MDASI-C for symptom assessment in this population (36). The scale demonstrated good reliability, validity, and utility, with a Cronbach’s alpha coefficient of 0.89 for the symptom severity items, indicating excellent internal consistency (28).

### Data analysis

Descriptive statistics were used to summarize the general data. Categorical data were described using frequencies and percentages. For continuous variables such as age, weight, and BMI, data were expressed as mean and standard deviation (mean ± SD). For data that did not follow a normal distribution, median and interquartile range (M [P25, P75]) were used.

Symptom networks were constructed using R packages such as “ggplot2,” “networktools,” “qgraph,” and “bootnet.” The symptom network diagram is constructed based on the EBICglasso function. The symptom network was constructed using the EBICglasso method, which combines L1 regularization with the extended Bayesian information criterion (EBIC) to filter out statistically insignificant weak associations and generate a sparse network structure. Setting the tuning parameter γ to 0.5 (range: 0–0.5) avoided overfitting. This method required no data transformation, thereby fully preserving the original dataset and its inherent information. The adjacency matrix generated by EBICglasso is natively compatible with R packages, facilitating subsequent centrality analysis and network stability validation (37). In these networks, symptoms are represented as nodes, and the lines between nodes are the edges. Thicker edges indicate stronger correlations between two symptoms (38).

To ensure the accuracy of core symptoms, edge weight bootstrapping and centrality stability tests were conducted (39). Centrality measures included strength, closeness, and betweenness. Strength is the sum of the absolute values of edge correlation coefficients and serves as an indicator of a node’s importance in the network. A higher value indicates that the symptom has a greater influence on other symptoms. Closeness is the reciprocal of the sum of distances between a node and all other nodes. A higher closeness value suggests that the symptom is closer to other symptoms and more likely to be central in the network. Betweenness measures the number of times a node lies on the shortest path between two other nodes. A higher betweenness value indicates that the symptom is more likely to act as a bridging symptom (38, 39). Additionally, the stability of centrality measures was tested by calculating the correlation stability (CS) coefficient, which should ideally be greater than 0.25 and preferably above 0.5. All statistical analyses were performed using IBM SPSS 25.0 and R software (version 4.4.2).

## Results

### Characteristics of participants

A total of 480 female breast cancer patients were included in this study, with ages ranging from 24 to 82 years (mean age: 52.46 ± 12.02 years). In terms of body mass index (BMI), 24 participants were underweight, 289 were of normal weight, 141 were overweight, and 23 were obese. Among the participants, 66 were from rural areas, while 411 were from urban areas. The majority of patients were in the later stages of chemotherapy (38.5%). (See **Table 1** for details).

**Table 1:** Characteristics of participants (n = 480).

Characteristics	n(%)	( $\bar{x}$ ± s)/M[P25,P75]
Age	\	52.46 ± 12.02
Weight(kg)	\	59.47 ± 8.47
Height(cm)	\	158.33 ± 20.46
BMI	\	22.97 ± 3.15
<18.5	22(4.6)	17.59 ± 0.79
18.5~23.9	289(60.2)	21.47 ± 1.46
24~27.9	141(29.4)	25.55 ± 1.14
>28	23(4.8)	30.77 ± 3.76
Educational background		
Primary school or below	38(7.9)	\
Junior high school	101(21)	\
Secondary school or high school	126(26.3)	\
College degree or above	201(41.9)	\
Marital status		
Be married	447(93.1)	\
Unmarried	23(4.8)	\
Divorce	4(0.8)	\
Be bereaved of one’s spouse	5(1)	\
Occupation		
Be on the job	152(31.7)	\
Retire	185(38.5)	\
Other	141(29.4)	\
Place of residence		
Village	66(13.8)	\
towns	411(85.6)	\
Medical insurance form		
self-financing	77(16)	\
Medical insurance	291(60.6)	\
Rural cooperative medical care	35(7.3)	\
other	69(14.4)	\
Chemotherapy stage		
Early chemotherapy cycles (cycle: 1~2)	79(16.5)	\
Middle chemotherapy cycles(cycle: 3~4)	164(34.2)	\
Late chemotherapy cycles (cycle >5)	185(38.5)	\

### Symptom prevalence, severity, and interference with daily life

This study investigated 13 symptoms in breast cancer patients. Among these symptoms, the most common were fatigue, sleep disturbance, and dry mouth, while the most severe were sleep disturbance, fatigue, and poor appetite. The symptoms with the highest prevalence of interference in daily life were work, mood, and general activity. Additionally, symptoms most significantly interfered with patients’ work, general activity, and mood. (See **Table 2** for details).

**Table 2:** Symptom prevalence and severity of participants (n = 480).

Symptoms	Prevalence:n(%)	Severity:(M[P25, P75], $\bar{x}$)
Symptom entry		
Fatigued	391(81.5)	3[1,5], 3.45
sleep disturbance	374(77.9)	3[1,6], 3.58
Dry mouth	352(73.3)	2[0,5], 2.77
Lack of appetite	346(72.1)	2[0,5], 2.92
Difficulty remembering	342(71.3)	2[0,5], 2.67
Distress	333(69.4)	2[0,4], 2.48
Drowsiness	330(68.8)	2[0,4], 2.51
Sadness	317(66)	1[0,3], 2.06
Nausea	295(61.5)	1[1,5], 2.61
Pain	285(59.4)	1[0,3], 2
Numbness	247(51.5)	1[0,3], 1.89
Vomit	244(50.8)	1[0,3], 1.97
Shortness of breath	238(49.6)	0[0,3], 1.63
Symptom interference item		
General activity	337(70.2)	2[0,5],2.83
Mood	349(72.7)	2[0,4],2.66
Work (including housework)	350(72.9)	2[0,6],3.3
Relationships with others	254(52.9)	1[0,2],1.58
Walking	240(50.0)	0.5[0,2],1.55
Enjoyment of life	308(64.2)	1[0,4],2.16
Total points		12[3, 21.75], 14.08

### Symptom networks

#### Overall network and residual network

**Figure 1:**
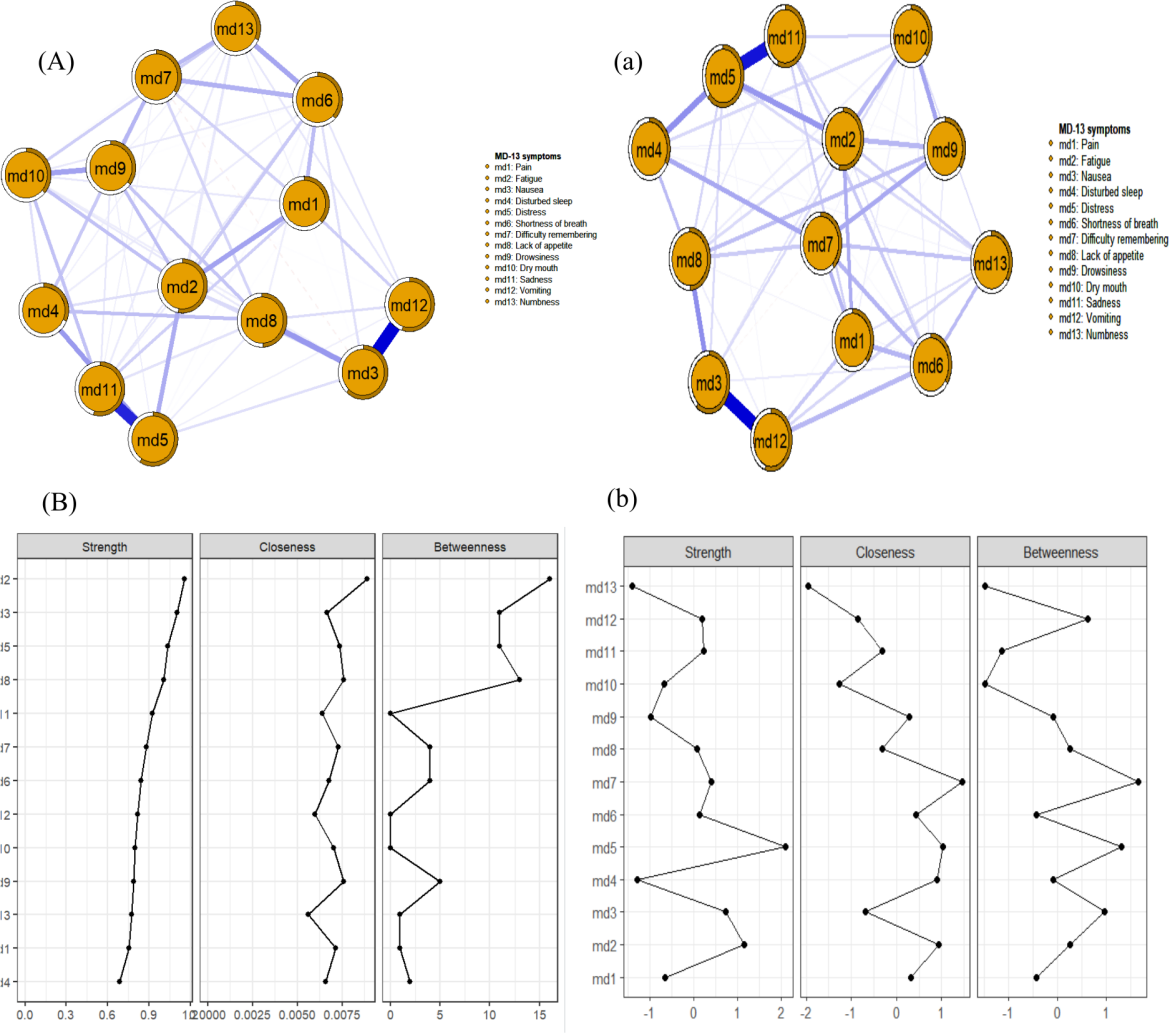
Overall and residual symptom networks, and centrality indices in breast cancer patients during chemotherapy.

**Figure 1A** displays the symptom network for breast cancer patients during chemotherapy. The core symptom in the network is fatigue. According to the Strength metric shown in **Figure 1B**, the top three symptoms are fatigue (Rs = 1.16), nausea (Rs = 1.11), and distress (Rs = 1.00). Predictability is represented by the circles around the nodes in **Figure 1A**, with node predictability values ranging from 31.7% to 61.7%. Nausea, distress, and vomiting have the highest predictability, indicating that 61.7%, 59.0%, and 55.0% of their variance can be explained by their neighboring symptoms. To exclude potential confounding effects of demographic and clinical factors, we constructed a residual symptom network with statistical control for variables including age, body mass index (BMI), and chemotherapy cycles, which was then compared with our original overall network (40). **Figure 1a** displays the residual symptom network. According to the Strength metric shown in **Figure 1b**, the top three symptoms remain fatigue (Rs = 1.05), nausea (Rs = 0.99), and distress (Rs = 1.19).

**Figure 2:**
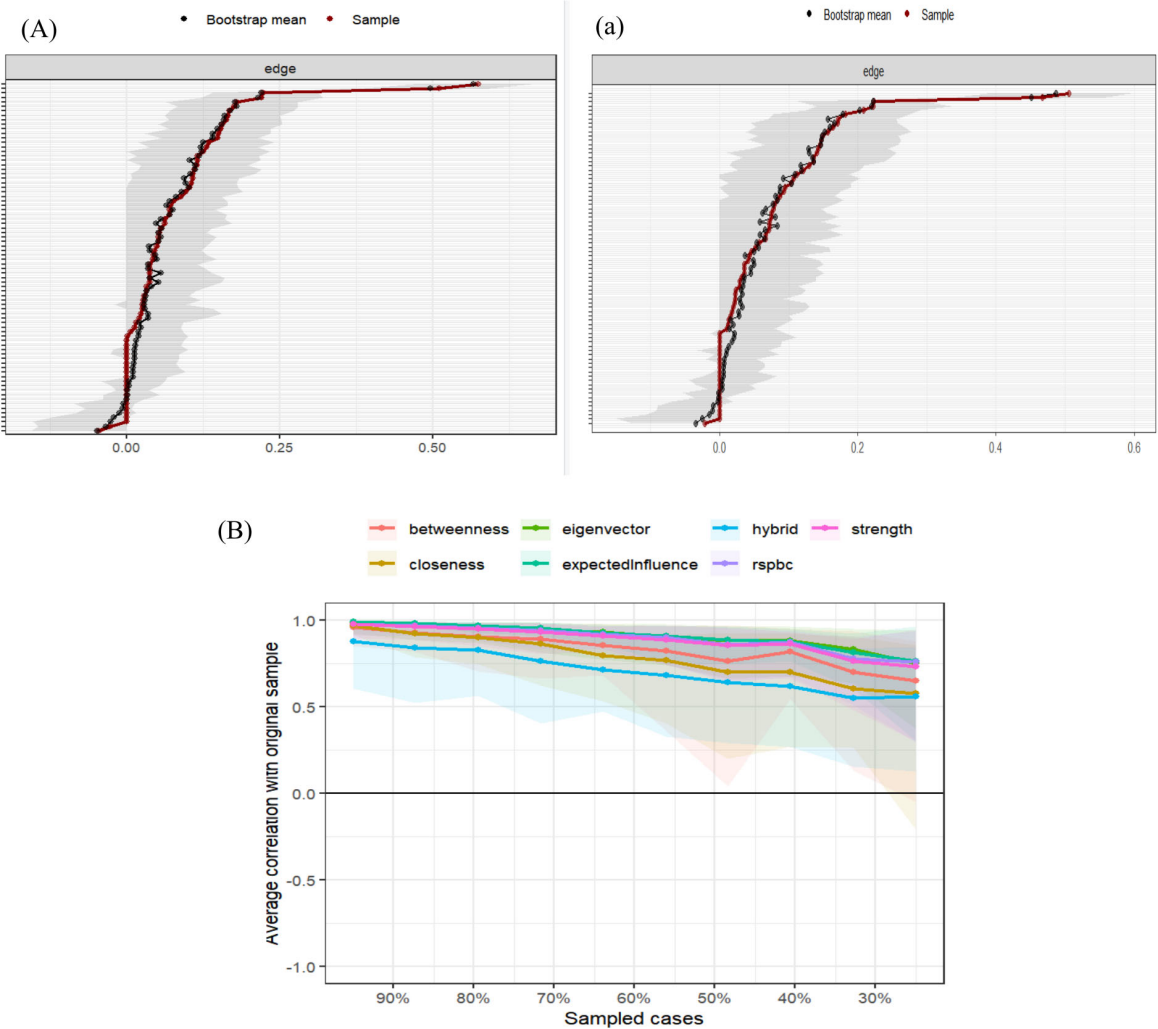
Accuracy Testing of Edge Weights and Stability Testing of Centrality in the Overall Symptom Network and Residual Symptom Network. **(A)** Edge weight accuracy test of the overall symptom network; **(a)** Edge weight accuracy test of the residual symptom network; **(B)** Centrality stability test of the overall symptom network.

**Figure 2A** displays the results of the edge weight bootstrap analysis. The bootstrap confidence intervals (CIs) are narrow, indicating good accuracy of the network. As the sample size decreases, the stability of centrality indices—betweenness, closeness, and strength—remains relatively high. The correlation stability (CS) coefficient results show that the values for betweenness (CS [cor = 0.7] = 0.36) and closeness (CS [cor = 0.7] = 0.283) are below 0.5 but above 0.25, indicating acceptable stability. The CS coefficient for strength centrality (CS [cor = 0.7] = 0.594) and expected influence (CS [cor = 0.7] = 0.594) exceeds 0.5, demonstrating good stability. **Figure 2a** displays the results of the residual network edge weight bootstrap analysis. The correlation stability (CS) coefficient results show that the values for betweenness (CS [cor = 0.7] = 0.205) and closeness (CS [cor = 0.7] = 0.205) are below 0.25. But the CS coefficient for strength centrality (CS [cor = 0.7] = 0.594) and expected influence (CS [cor = 0.7] = 0.671) exceeds 0.5, demonstrating good stability. (See **Table 3** for details).

**Table 3:** Overall and subgroup symptom network CS coefficients.

Network type	Subgroup	Strength CS (Assessment)	Betweenness CS (Assessment)	Closeness CS (Assessment)	Expected influence CS (Assessment)
Overall Network		0.594 (Good)	0.360 (Acceptable)	0.283 (Acceptable)	0.594 (Good)
Network Comparison by BMI Categories	Normal	0.361 (Acceptable)	0.052 (Unstable)	0.206 (Unstable)	0.438 (Acceptable)
	Abnormal	0.284 (Acceptable)	0.051 (Unstable)	0.126 (Unstable)	0.284 (Acceptable)
Network Comparison by Chemotherapy Cycle	Cycle 1	0.282 (Acceptable)	0.282 (Acceptable)	0.208 (Unstable)	0.282 (Acceptable)
	Cycle 2	0.359 (Acceptable)	0.131 (Unstable)	0.207 (Unstable)	0.359 (Acceptable)
	Cycle 3	0.284 (Acceptable)	0 (Unstable)	0.052 (Unstable)	0.284 (Acceptable)

Stability criteria: ≥ 0.50 = Good stability; 0.25 ≤ CS<0.50 = Acceptable stability; <0.25 = Unstable.

Cycle 1=Early chemotherapy cycles; Cycle 2=Middle chemotherapy cycles; Cycle 3=Late chemotherapy cycles.

**Figure 3:**
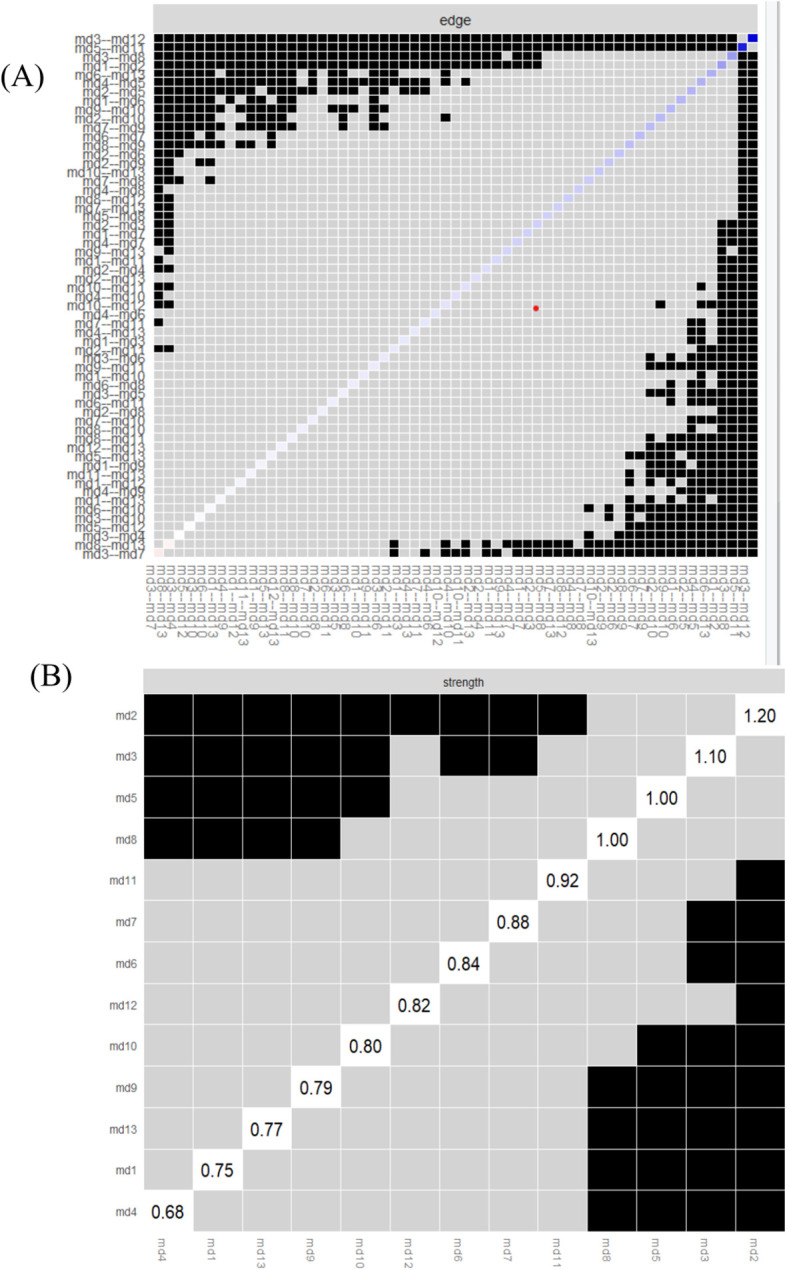
Edge weight difference test **(A)** and bootstrapped node difference test **(B)** results of the symptom network in breast cancer patients during chemotherapy.

**Figure 3A** shows the results of the edge weight difference test. The bootstrap difference test for edge weights indicates that the edge weights between md3 (nausea) and md12 (vomiting), as well as between md5 (distress) and md11 (sadness), are significantly different from approximately 95% of the other edge weights in the network. **Figure 3B** displays the results of the bootstrapped node difference test. The strength of most nodes does not differ significantly from one another. md2 (fatigue) is significantly different from other nodes (Difference Test Strength [DTs] = 1.20).

### Subgroup analysis

#### Network comparison by BMI categories

**Figure 4:**
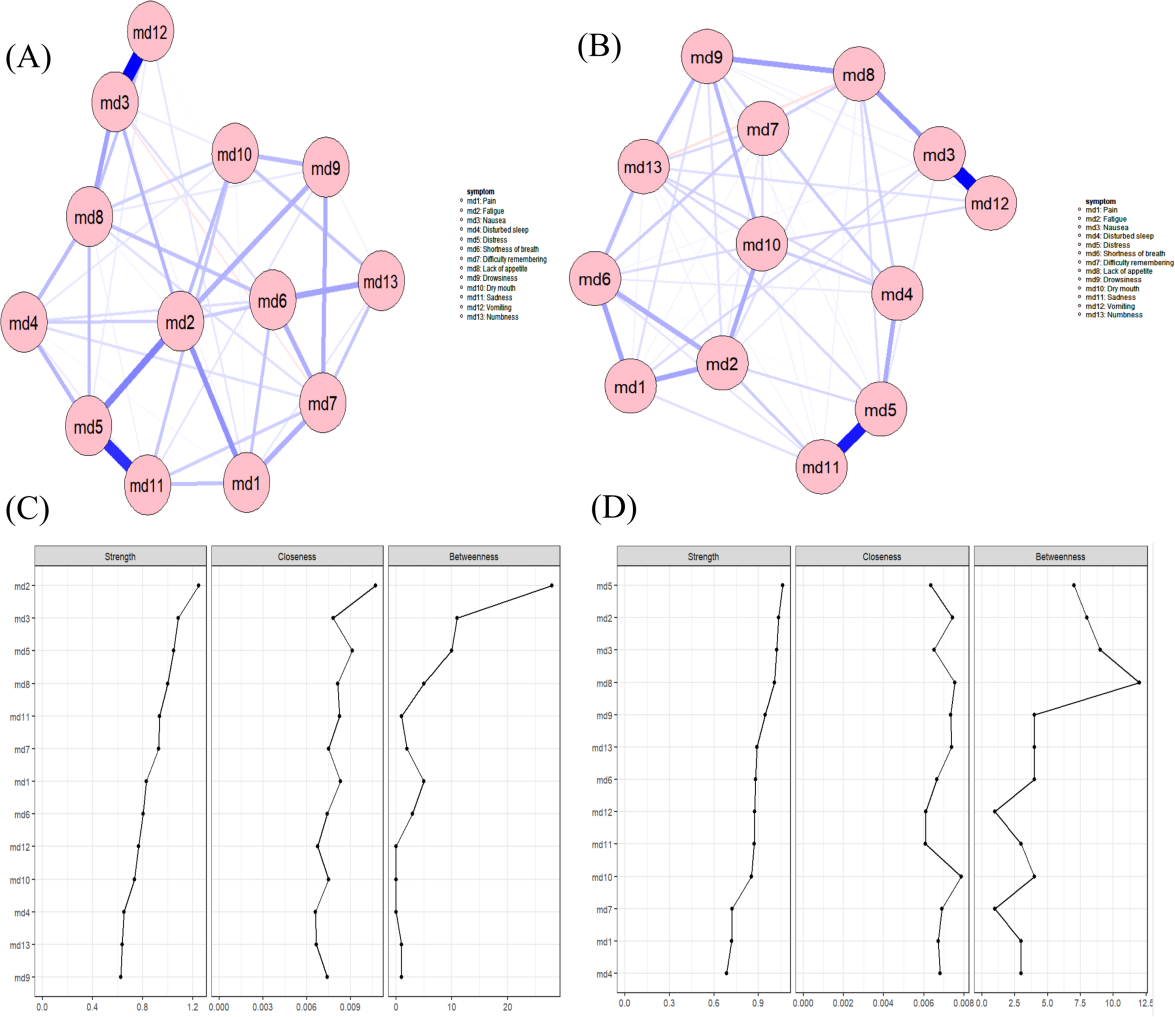
**(A)** Symptom network of the normal BMI subgroup; **(B)** Symptom network of the high BMI subgroup; **(C)** Centrality indicators of the symptom network in the normal BMI subgroup; **(D)** Centrality indicators of the symptom network in the high BMI subgroup.

While multiple linear regression analysis revealed no significant effects of BMI or chemotherapy cycles on overall symptom severity, prior research has established that both factors can drive alterations in symptom profiles (41, 42). We therefore constructed stratified symptom networks by BMI and chemotherapy cycle to further explore these nuanced relationships. Based on the thickness of the edges in the symptom networks, **Figure 4A** shows that in both the BMI (normal BMI group, BMI:18.5~23.9) and high BMI (Overweight or obese group, BMI>24) subgroups, the symptom pairs with positive and strong correlations are md3 (nausea) and md12 (vomiting), as well as md5 (distress) and md11 (sadness). In the BMI group, the core symptom in the network is md2 (fatigue), while in the high BMI group, the core symptom is md5 (distress). (See **Figure 4** for details.). The correlation stability (CS) coefficient results for the normal BMI group show that the values for betweenness (CS [cor = 0.7] = 0.052) and closeness (CS [cor = 0.7] = 0.206) are below 0.25, indicating unstable stability. The CS coefficient for strength centrality (CS [cor = 0.7] = 0.361) and expected influence (CS [cor = 0.7] = 0.438) are below 0.5 but above 0.25, demonstrating acceptable stability. For the abnormal BMI group, betweenness (CS [cor = 0.7] = 0.051) and closeness (CS [cor = 0.7] = 0.126) also showed unstable stability (CS < 0.25), while strength centrality (CS [cor = 0.7] = 0.284) and expected influence (CS [cor = 0.7] = 0.284) achieved acceptable stability (0.25 < CS < 0.5). (See **Table 3** for details.).

#### Network comparison by chemotherapy cycle

**Figure 5:**
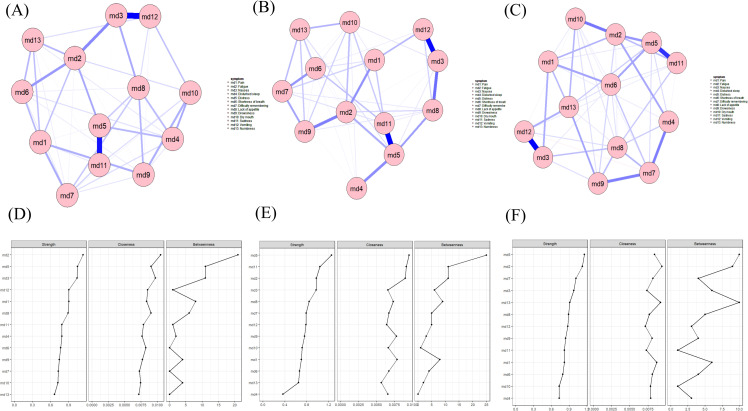
**(A)** Symptom network of the early chemotherapy subgroup; **(B)** Symptom network of the middle chemotherapy subgroup; **(C)** Symptom network of the late chemotherapy subgroup; **(D)** Centrality indicators of the symptom network in the early chemotherapy subgroup; **(E)** Centrality indicators of the symptom network in the middle chemotherapy subgroup; **(F)** Centrality indicators of the symptom network in the late chemotherapy subgroup.

Based on the thickness of the edges in the symptom networks, the symptom pairs with positive and strong correlations in the early chemotherapy subgroup are md3 (nausea) and md12 (vomiting), as well as md5 (distress) and md11 (sadness). According to the strength centrality metric, the core symptom in the symptom network during the pre-chemotherapy phase is consistently md2 (fatigue), while the core symptoms during the mid-chemotherapy and post-chemotherapy phases are md5 (distress). (See **Figure 5** for details.). The correlation stability (CS) coefficient results for the early chemotherapy group show that the values for closeness (CS [cor = 0.7] = 0.208) are below 0.25, indicating unstable stability. The CS coefficient for strength centrality (CS [cor = 0.7] = 0.282) and betweenness (CS [cor = 0.7] = 0.282) are below 0.5 but above 0.25, demonstrating acceptable stability. The stability coefficient (CS) results for the middle chemotherapy and late chemotherapy groups indicate that the strength-centrality CS coefficient (CS[cor=0.7]=0.361) are below 0.5 but above 0.25, suggesting its stability falls within an acceptable range. Both the closeness and betweenness CS coefficients are below 0.25. (See **Table 3** for details.).

#### Relationship between symptoms and daily living status

**Figure 6:**
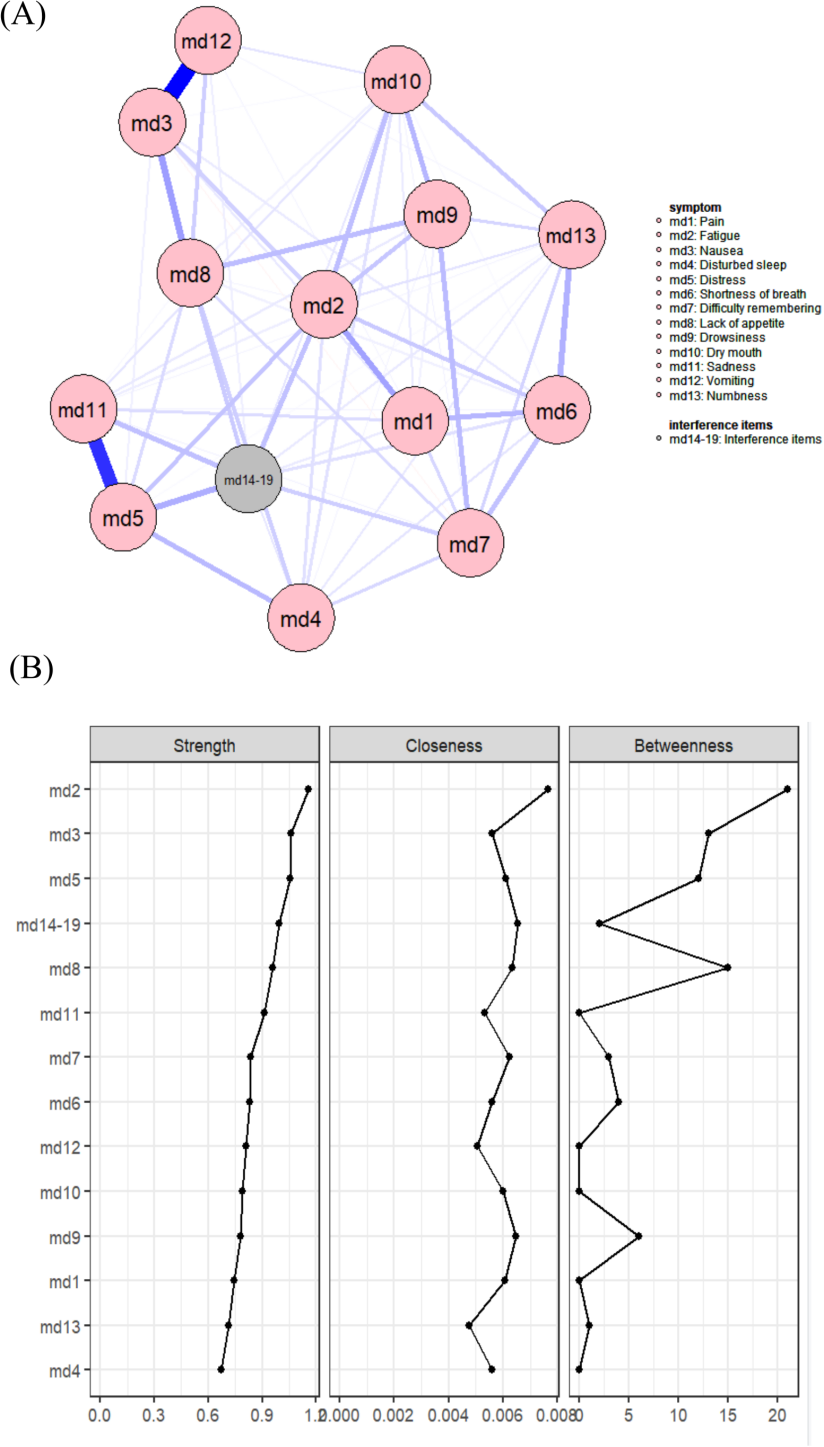
Symptom Network and Centrality Indicators of Symptoms and Interference Items. **(A)** Symptom network of symptoms and interference items; **(B)** Centrality indicators of the symptom network for symptoms and interference items.

Fatigue is the most central node and shows a positive correlation with the total score of daily life interference. The symptom closest to the total score of daily life interference and with the thickest edge is distress. (See **Figure 6** for details).

**Figure 7:**
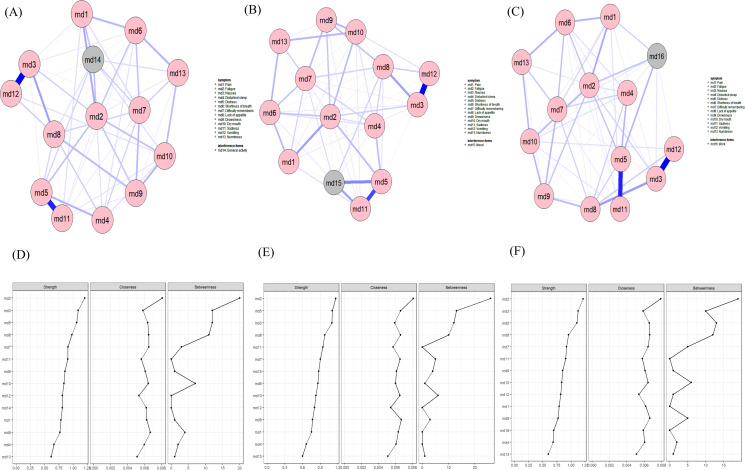
Symptom networks and centrality indicators of symptoms relative to various interference items **(A)** Symptom network of symptoms and general activity; **(B)** Symptom network of symptoms and mood; **(C)** Symptom network of symptoms and work; **(D)** Centrality indicators of symptoms in the general activity symptom network; **(E)** Centrality indicators of symptoms in the mood symptom network; **(F)** Centrality indicators of symptoms in the work symptom network.

Fatigue is the most central symptom across the three networks in **Figure 7**, showing a positive correlation with general activity and work. The symptoms closest to general activity and with the thickest edges are pain and fatigue. Distress and sadness have the highest proximity and edge thickness with mood, while the symptoms that most significantly impact patients’ work are fatigue and distress. (See **Figure 6** for details).

## Discussion

Our study found that among breast cancer patients during chemotherapy, the symptoms with the highest prevalence were fatigue, sleep disorders, and dry mouth, while the most severe symptoms were sleep disturbance, fatigue, and lack of appetite. Fatigue is regarded as the most distressing and prevalent symptom experienced by breast cancer patients (43), especially those undergoing chemotherapy (44). The core symptoms identified in this study are associated across different cancer types: fatigue and psychological distress were both confirmed as core symptoms in the chemotherapy symptom network of breast cancer patients (consistent across overall and subgroup analyses), aligning with the general characteristics of chemotherapy-related symptom networks. These symptoms also form the core of chemotherapy symptom networks in lung cancer patients (45) and are particularly prominent in gynecologic cancer cohorts (where fatigue is a core symptom and both exhibit the most severe severity) (46), highlighting their critical impact on cancer patients’ functional status and their value as priority intervention targets for precision chemotherapy symptom management. Despite having access to data on the prevalence and severity of symptoms, understanding the mechanisms underlying symptom exacerbation and relief remains a challenge. Network analysis offers a promising approach, enabling researchers to identify key symptoms and uncover their interrelationships (47). This, in turn, allows for the identification of potentially more core symptoms from a mechanistic standpoint. Previous studies have commonly identified fatigue as the most central symptom among cancer patient (7, 9), which aligns with the findings of the present study. To verify the robustness of our findings, correlation stability (CS) analysis was performed on both the overall and subgroup symptom networks. Among network metrics, strength centrality and expected influence (EI) are recognized as the most critical due to their generally high CS coefficients and strong interpretability. Strength centrality reflects the total connection weight of a node, directly indicating its network importance (39), while EI comprehensively captures both direct and indirect node effects (48). In contrast, betweenness and closeness centrality typically show low stability and are rarely used as primary analytical metrics. Our results demonstrated acceptable stability for both strength centrality and EI (CS > 0.25) across the overall and subgroup networks, validating the reliability of our core findings. Based on a stability-validated analytical framework, the network analysis conducted in this research further reveals that during the entire chemotherapy period for breast cancer patients, fatigue (Rs = 1.16), nausea (Rs = 1.11), and distress (Rs = 1.00) emerge as the core symptoms. In contrast, sleep disorders, dry mouth, and poor appetite do not hold this central position. These results suggest that a high incidence and severity of symptoms do not automatically equate to a symptom being core for patients. The core symptoms identified through network analysis may reflect deeper underlying pathophysiological or psychological mechanisms that are crucial for targeted intervention and patient care. Based on the predictability results within the overall network, nausea, distress, and vomiting exhibited the highest predictability. This suggests that these symptoms may be located at the center of the network and are predicted by other symptoms within it. Therefore, when managing patients’ symptoms, a comprehensive consideration of the patients’ overall symptom profiles should be taken into account, rather than merely focusing on the symptoms with the highest incidence and severity. Simultaneously, attention should be paid to the relationships between symptoms. For instance, if one aims to alleviate a patient’s nausea symptoms, considering that nausea symptoms are highly predictable, precise interventions can be carried out on the symptoms closely associated with them.

To further explore the heterogeneity of symptom networks, subgroup analyses were conducted based on BMI categories and chemotherapy cycle. When conducting a subgroup analysis, a nuanced pattern emerged. Specifically, in the early chemotherapy cycles, the most prominent symptoms were fatigue, distress, and nausea. Moving on to the middle chemotherapy cycles, the focus shifted to distress, sadness, and fatigue as the core symptoms. Finally, in the late chemotherapy cycles, distress, fatigue, and forgetfulness took center stage. This progressive change indicates that as the chemotherapy process unfolds, patients’ psychological issues gradually assume a more prominent and dominant role. This finding aligns closely with the results of Rha and Lee’s study (9). In that research, the longitudinal data of 249 cancer patients undergoing chemotherapy were meticulously analyzed, revealing that the centrality of fatigue diminished after the fourth chemotherapy cycle. Moreover, distress, sadness, and loss of appetite were found to be the core symptoms among cancer survivors. This parallelism not only validates our current findings but also implies that psychological problems exert a profound and enduring influence on breast cancer patients throughout their extended treatment journey. Individuals diagnosed with cancer frequently encounter a spectrum of psychological challenges, encompassing distress, anxiety, and depression (49). These psychological burdens are intricately linked to various factors unique to breast cancer patients, such as their struggle to accept alterations in self - image, difficulties in social adaptation, and the ever - looming fear of recurrence. Given the far - reaching implications of these psychological issues, addressing psychological distress becomes an imperative. Not only do these symptoms significantly impact the patient’s quality of life (50), but they can also act as a deterrent to treatment completion. Consequently, this has a detrimental effect on the disease prognosis and the likelihood of recurrence. Thus, it becomes evident that early detection and effective alleviation of emotional distress through appropriate psychological adjustment strategies should be an integral and indispensable part of long - term symptom management.

In addition to the psychological aspects, a notable trend was observed when comparing the symptom networks across the three chemotherapy cycles. Specifically, the cognitive impairment among breast cancer patients showed a progressive deterioration, eventually emerging as one of the core symptoms in the late stage of chemotherapy. The incidence of chemotherapy - related cognitive impairment in breast cancer patients spans a wide range, from 16% to 75%. Approximately 35% of patients experience a sustained decline in cognitive function in the years following chemotherapy (51). This cognitive impairment not only undermines treatment compliance and diminishes the quality of life of breast cancer (BC) patients, thereby escalating the overall disease burden but also impairs their perception of social roles and restricts their active engagement in social activities (52). In light of these consequences, it is clear that in the late stage of chemotherapy, the prevention and treatment of cognitive impairment should be elevated to a position of high priority within the framework of symptom management.

Our study found that the core symptoms of breast cancer patients during chemotherapy differed between the normal BMI subgroup and the high BMI subgroup. For patients with normal BMI, the core symptoms were fatigue, nausea, and distress. In contrast, for those with abnormal BMI, the core symptoms were distress, fatigue, and nausea. The study by Ng B et al. (53) showed that patients with a low or normal body mass index (BMI) were significantly associated with an increased risk of nausea and vomiting related to the first - cycle chemotherapy. Moreover, nausea and vomiting can exacerbate patients’ fatigue. According to the obesity paradox, obesity may have a protective effect on patients (54), reducing the physiological symptoms during chemotherapy. This could be one of the reasons for the discrepancy in the core symptoms between the two subgroups. Compared with patients with normal BMI, overweight or obese patients have more needs and concerns regarding weight management, which may lead to more psychological problems. The mechanism by which BMI affects the core symptoms of patients remains unclear. Therefore, further research is needed to explore the impact of BMI on core symptoms. Additionally, different intervention strategies should be adopted for patients with different BMIs. For patients with normal BMI, more attention should be paid to their physiological symptoms, for example, nutritional support and physical exercise interventions for overweight or obese patients, however, greater emphasis should be placed on their psychological symptoms, and interventions should be targeted at the causes of their psychological and emotional changes.

In the present study, we found that work, mood, and general activities were the aspects of daily life most significantly impacted by symptoms. This finding aligns with the results of a study conducted by Zhu et al. (30), which reported that general activities and mood were the most affected daily - life domains among breast cancer patients undergoing chemotherapy. To further explore these relationships, we incorporated the total score of daily - life interference, along with scores for general activities, mood, and work, into a symptom network analysis. The results indicated that fatigue and distress were the symptoms most closely associated with overall daily - life interference. Specifically, pain and fatigue had the greatest influence on general activities; distress and sadness were the primary factors affecting mood; and fatigue and distress were the most impactful on work. These findings underscore the substantial influence of fatigue and psychological issues on patients’ daily lives. A qualitative study by L’Hotta et al. (31) on cancer patients’ perspectives regarding daily - life participation revealed that patients often experience a sense of helplessness and a loss of control over their daily routines, which hinders their ability to engage in normal daily activities. Moreover, Ciria - Suarez’s research (55) demonstrated that breast cancer patients aspire to shed the “patient” label. By minimizing the impact of symptoms on daily life, we can potentially enable patients to resume a normal lifestyle, thereby reducing their self - perception as patients and alleviating misunderstandings from others. Given these insights, in addition to implementing interventions aimed at reducing the overall symptom burden, it is crucial to specifically target those symptoms that have a disproportionate impact on daily life. This approach can effectively support patients in their journey towards reintegrating into normal life. Our study represents one of the limited investigations that have employed symptom network analysis to explore the intricate relationship between symptoms and daily life. By doing so, it not only offers valuable insights for symptom management but also addresses an existing gap in the literature, contributing to a more comprehensive understanding of how symptoms affect the daily lives of breast cancer patients.

This study provides innovative contributions to chemotherapy-related symptom network research in breast cancer patients, filling gaps in existing literature. First, it examines how symptom network characteristics influence patients’ daily living conditions, overcoming prior studies’ focus solely on symptom correlations (56). Linking network features to daily outcomes offers actionable evidence for targeted quality-of-life interventions. Second, multidimensional subgroup analyses (by BMI and chemotherapy cycles) identify network heterogeneity and dynamic changes, advancing prior univariate subgroup studies by clarifying structural differences across subgroups (57). Third, we established a systematic framework integrating holistic network construction, subgroup analysis, and clinical outcome assessment, addressing prior fragmentation and enabling multi-level symptom exploration to support precision clinical symptom management.

## Limitations

Due to the lack of specific hospital information in the original dataset, this secondary analysis was unable to assess potential inter-center differences and clustering effects. Future studies may further investigate center-related variability and clustering effects through stratified analysis by research center. Our study was not a longitudinal one. As a result, we were unable to analyze how the symptoms of breast cancer patients changed over chemotherapy cycles, nor could we determine the causal relationships among symptoms. However, we explored the symptom differences among patients at different chemotherapy stages, and the research findings were similar to those of existing longitudinal studies. In addition, patients with severe comorbidities were excluded to minimize confounding of chemotherapy-related symptom networks, which may alter the centrality of specific symptoms by lowering that of somatic hub symptoms and simplifying network structure. Thus, the assessed symptom severity and centrality may not be representative of the overall breast cancer population undergoing chemotherapy. Finally, the networks generated from our subgroup analyses exhibited insufficient stability. This instability is likely attributable to the relatively small sample size within each subgroup, which may have obscured the true associations between symptoms and rendered the inter-symptom relationships less definitive and robust. Consequently, future investigations with larger, adequately powered samples are warranted to conduct subgroup network analyses.

## Conclusion

This study identified the symptom networks of 480 breast cancer patients undergoing chemotherapy, encompassing overall symptoms, different chemotherapy phases, and varying BMI subgroups. Our findings reveal that fatigue is the most central symptom in the overall symptom network, the BMI subgroup, and the early chemotherapy cycles. In contrast, distress emerges as the most central symptom in the middle chemotherapy cycles, late chemotherapy cycles, and high BMI subgroups. Distress also has the greatest impact on daily life functioning. Assessing the interference of symptoms with daily life can help optimize symptom management strategies for breast cancer patients receiving chemotherapy, aiding them in restoring normal daily functioning. These findings provide valuable insights for continuously refining symptom management strategies for breast cancer patients during chemotherapy.

## Data Availability

The raw data supporting the conclusions of this article will be made available by the authors, without undue reservation.
